# Prostate Cancer Development Is Not Affected by Statin Use in Patients with Elevated PSA Levels

**DOI:** 10.3390/cancers11070953

**Published:** 2019-07-07

**Authors:** Dennie Meijer, R. Jeroen A. van Moorselaar, André N. Vis, Irene V. Bijnsdorp

**Affiliations:** Department of Urology, Amsterdam UMC, Vrije Universiteit Amsterdam, Cancer Center Amsterdam, De Boelelaan 1117, 1081HV Amsterdam, The Netherlands

**Keywords:** statin, biochemical recurrence, biochemically recurrent, prostate cancer

## Abstract

*Background*: The role of statins in prostate cancer (PCa) remains unclear. Conflicting evidence has been found concerning risk reduction with the use of statins on biochemical recurrence (BCR). In this study, we evaluated whether statin use decreases the incidence of advanced PCa in males with elevated prostate-specific antigen (PSA; ≥4.0 ng/mL) levels and determined whether statin use reduces the risk of BCR after radical prostatectomy (RP). *Methods*: Patients visiting the outpatient urology clinic of the VU Medical Center between 2006 and 2018 with elevated PSA were retrospectively analyzed. Biochemical recurrence after RP was defined as a PSA level of ≥0.2 ng/mL (measured twice). *Results*: A total of 1566 patients were included, of which 1122 (72%) were diagnosed with PCa. At the time of diagnosis, 252 patients (23%) used statins compared to 83 patients (19%) in the non-malignancy group (*p* = 0.10). No differences were found in the use of statins between the different risk groups. No correlation was found between the risk of BCR after RP and the use of statins in the total (*p* = 0.20), the intermediate-risk group (*p* = 0.63) or the high-risk group (*p* = 0.14). *Conclusion*: The use of statins does not affect PCa development/progression in patients with elevated PSA levels, nor the development of BCR after RP.

## 1. Introduction

Prostate cancer (PCa) is the most common cancer in males in the western world and the worldwide burden of this malignancy is rising [1,2]. Frequently, PCa is detected after an initial measurement of elevated prostate-specific antigen (PSA) levels. Evidence for several modifiable risk factors is still uncertain, however, lifestyle, such as smoking and exercise, can influence the risk of developing PCa [3,4,5,6].

Statins are widely used for the treatment of hypercholesterolemia. In addition to reducing cholesterol levels, statins have a number of other beneficial effects that provide new possibilities for the use of statins. One of the described mechanisms to prevent PCa (progression) is by the induction of apoptosis and cell growth arrest [7]. Several studies examined the risk reduction of statins in PCa patients, though conflicting results were observed [8,9]. A meta-analysis among thirteen observational studies and six randomized controlled trials showed that long-term statin use did not affect the risk of developing PCa. Nonetheless, a significantly reduced incidence of advanced PCa was observed [10]. The studies that contributed the most to this meta-analysis compared patients who used statins at any time with patients who had never used statins, instead of evaluating the patients that were using statins at the time of diagnosis [11,12]. For the two studies that did examine statin use at the time of diagnosis, conflicting evidence was reported [13,14]. 

Statins may also decrease the risk of biochemical recurrence (BCR) in PCa patients that received active treatment [15]. The definition of BCR is two consecutive PSA values ≥0.2 ng/mL after a radical prostatectomy (RP), or any PSA level ≥2.0 ng/mL above the nadir after external beam radiation therapy or brachytherapy [16,17,18]. A meta-analysis of five observational studies showed no significant difference between the risk of BCR in statin users versus in non-statin users in patients after RP [19], whereas a systematic review showed that the use of statins lowers the chance of developing BCR [20]. However, to date, the effect of statin use on PCa progression and BCR in patients with elevated PSA levels (≥4.0 ng/mL) has not been studied. Whether statin subscription in these patients can prevent PCa progression or BCR is unknown. Therefore, the aim of this study was to determine the possible correlation between statins, the presence of PCa, and BCR in males with elevated levels of PSA.

## 2. Results

**Figure 1:**
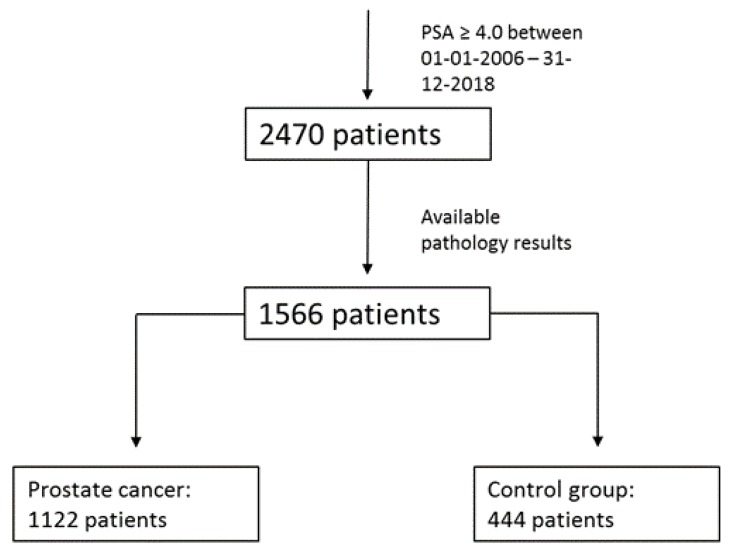
Flowchart including patients.

Out of the 2470 consecutive patients who visited the outpatient urology clinic, 1566 patients were included in this study (Figure 1). The clinical characteristics are presented in Table 1. Among the subjects, 1122 (71.6%) patients were diagnosed with PCa. The ages between the control and the PCa patients at the time of diagnosis were similar (*p* = 0.24). The percentage of statin users was 22.5% in the PCa group compared to 18.7% in the control group (*p* = 0.10). The body mass index (BMI) was significantly higher in the PCa group compared to the control group *(p* = 0.01), while the percentage of statin users in obese patients (BMI ≥ 30 kg/m^2^) was slightly higher (33.3%) compared to patients with a BMI < 30 kg/m^2^ (26.2%; *p* = 0.05). Furthermore, in overweight patients (BMI ≥ 25 kg/m^2^) the percentage of statin users was significantly higher (*p* = 0.001) compared to patients with a BMI < 25 kg/m^2^ (Table 2). Since we only included patients with elevated levels of PSA, the first PSA measurement was not a valuable factor for stratifying patients into the different groups (*p* = 0.93). Body mass index was evaluated among the different risk groups. 

Comparative analyses were performed to evaluate BMI between the different risk groups, specifically low risk vs. intermediate risk, low risk vs. high risk, and intermediate risk vs. high risk. These analyses showed no statistically significant difference (Table 2). 

The use of statins was similar between the patients in the different risk groups *(p* = non-significant; Table 2). This indicates that there is no effect of statin use on the primary development and progression of PCa in patients with elevated PSA levels. 

The ages of the patients did not differ significantly between the PCa group and the control group in patients with BMI < 30 kg/m^2^ (*p* = 0.20), nor in patients with BMI ≥ 30 kg/m^2^ (*p* = 0.19). Moreover, in patients using statins, no difference in age was evaluated (*p* = 0.37; Table 3), indicating that neither statin use nor BMI influences the time of diagnosis of PCa.

**Figure 2:**
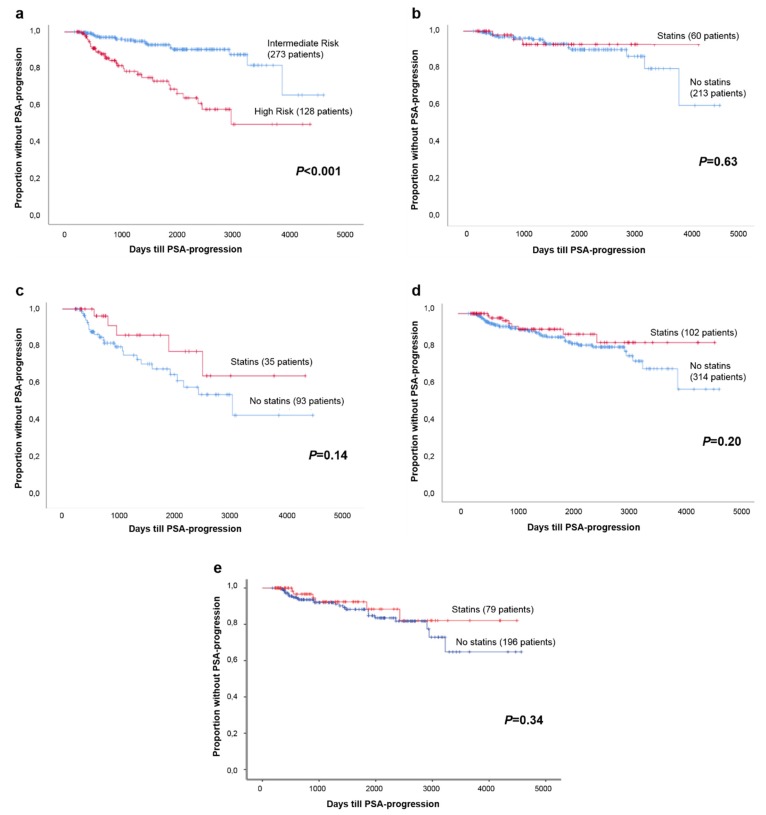
(**a**) Survival analysis; chance of developing BCR between intermediate-risk group and high-risk group; (**b**) Survival analysis; chance of developing BCR between statin users and non-statin users; (**c**) Survival analysis; chance of developing BCR between statin users and non-statin users in the intermediate-risk group; (**d**) Survival analysis; chance of developing BCR between statin users and non-statin users in the high-risk group; (**e**) Survival analysis; chance of developing BCR between statin users and non-statin users in the BMI ≥ 25 kg/m^2^ group.

Patients classified as high risk showed a significantly increased risk of developing BCR after RP (*p* < 0.001; Figure 2a). To determine the effect of statin use on BCR in patients that underwent RP, survival analyses were performed. Biochemical recurrence was found in 7.0% of the patients with PCa. Patients with residual prostate tissue after surgery or without adequate follow-up were excluded from these analyses.

In all the patients that underwent RP, statin use did not influence the risk of developing BCR (*p* = 0.20; Figure 2b). We evaluated whether statin use affected BCR in the different risk groups, as well as in the group with overweight patients (BMI ≥ 25 kg/m^2^). The intermediate-risk and high-risk group survival analyses are presented in Figure 2c and Figure 2d, respectively. In both categories, no difference was found between statin users and non-statin users and the chance of developing BCR (*p* = 0.14 and *p* = 0.20, respectively). In Figure 2e, the survival analysis of patients with a BMI ≥ 25 kg/m^2^ is shown. No statistically significant difference was found between statin users and non-statin users and the chance of developing BCR (*p* = 0.34).

## 3. Discussion

In this retrospective study, we evaluated 2470 patients that visited the outpatient urology clinic with an elevated PSA. Consequently, 1566 patients received a definite diagnosis, confirmed with a histopathological examination. We aimed to generate evidence on the association between statin use and clinical outcomes among a large group of males that presented elevated levels of PSA. Currently, there are no guidelines as to the use of statin medications in the prevention of PCa progression or BCR for patients that have elevated levels of PSA. To our knowledge, this is the first large-scale study evaluating statins in patients with elevated PSA. 

In our cohort, only a minority of PCa patients was classified as low risk (9.3%). Several studies showed that patients who used statins had lower PSA levels than patients who had never used statins, suggesting that this might lead to a later detection of PCa in these patients [21,22,23]. As we only included patients with elevated PSA levels, we might have detected these patients in a later stadium, resulting in the low percentage of patients classified as low risk. However, no significant difference in the distribution of patients among the different risk groups between statin users and non-statin users was evaluated. Therefore, the clinical significance of this hypothesis remains unclear.

In our cohort, no difference between the patients that were diagnosed with PCa and the number of patients that used statins was observed. The percentage of patients that used statins at the time of diagnosis is in agreement with the use of statins in the Netherlands in the general male population aged 45–74 years, which was estimated at around 20% in 2017 [24,25]. This is comparable with both the PCa group and the control group in our cohort. Therefore, patients with elevated levels of PSA are unlikely to use statins more or less frequent compared to the general population. 

In the current literature, statins are frequently associated with a possible risk reduction for developing advanced PCa in the male population. Platz et al. [13] reported that the use of statins is not associated with the risk of PCa development overall but that it is associated with a reduced risk of advanced (especially metastatic or fatal) PCa. In contrast, Caro-Maldonado et al. [26] reported that low-dose statin treatment increases PCa aggressiveness in an in vitro analysis. Due to this conflicting evidence, we evaluated statin use amongst a large cohort (n = 1566) of patients with elevated PSA. We found that statin use did not differ between the patients diagnosed with cancer, nor with the aggressiveness of the diagnosed cancer.

The potential relationship between BMI and PCa risk has been evaluated in multiple cohort studies. A recent meta-analysis showed that high BMI might be related to an increased risk of developing advanced PCa [27]. Obesity leads to high circulating concentrations of different hormones, such as insulin and leptin and low levels of the hormone adiponectin, all factors which have been described to increase the risk of advanced PCa [28]. Furthermore, obese patients frequently have elevated levels of blood cholesterol, which is a precursor for testosterone [29]. A review of PCa and cholesterol found that hypercholesterolemia is a reasonable risk factor for developing advanced PCa [30]. Unfortunately, recently reported studies that showed the association between BMI and advanced PCa did not take the use of lipid-lowering drugs into account [27,28]. In our study, we found that BMI was not a valuable factor to stratify between the different risk groups. This can possibly be explained by the fact that the percentage of patients with an elevated BMI (both ≥25 kg/m^2^ and ≥30 kg/m^2^) using statins was significantly higher compared to patients with a lower BMI (<25 kg/m^2^ and <30 kg/m^2^). 

According to the current literature, of the patients who underwent RP with curative intent, 20–40% developed BCR [31,32,33,34]. As the proportion of patients developing BCR after RP is rather high, several studies have been performed evaluating possible therapies to lower this percentage. There is increasing interest in the potential ability of statins to improve PCa outcomes and decrease BCR risk. One of these studies showed approximately 12% risk reduction of BCR in statin users compared with non-statin users [35]. Although this effect seems promising, the study was performed by evaluating all the different treatment modalities. When analyzed by treatment modality, statin use had a neutral effect on BCR among men who underwent RP. We also found that statin use in general was not associated with a reduced risk of BCR after RP. This is in agreement with the study of Ishak-Howard et al. [36], which showed that, in a small cohort of 539 patients, there was no association between statin use and the risk of developing BCR after RP. Although non-significant, our results in the intermediate- and high-risk groups showed a small trend towards the possible risk reduction of statin use on the development of BCR after RP. 

One of the limitations in the retrospective studies was the lack of information about the compliance of patients with prescribed statins, or changes in medication. Furthermore, due to the fact that statins are frequently prescribed by the general practitioner and we do not have access to these electronic patient charts, appropriate analysis of the duration of statin use is not possible. Therefore, no causality between the length of exposure to statins and PCa progression, nor with BCR in patients with elevated levels of PSA could be established.

Our results showed that statins do not seem to affect PCa progression and BCR in patients that have elevated levels of PSA. Therefore, prescribing statins to patients in this specific group might not be effective. The effect of specific types of statins on PCa progression and BCR should be studied in large prospective trials with controlled medications. 

## 4. Material and Methods

We retrospectively analyzed patients with elevated PSA (≥4.0 ng/mL) who visited the outpatient clinic of the department of Urology at the Amsterdam UMC, at the location of the VU Medical Center (VUmc), a large tertiary center. Patients between 2006 and 2018 were included for analysis. Patients without any available tissue histology results (e.g., biopsy or surgery) were excluded from the analysis. Patients with PCa were classified as low, intermediate and high risk, based on the ESMO-classification [37]. Biochemical recurrence after RP was defined as a PSA level of ≥0.2 ng/mL (measured twice). Patient data were collected from the electronic patient records. The obtained clinical information included age, Gleason score [38], TNM classification [39], PSA levels, imaging outcomes, previous treatment, use of medication and management decisions. Statistical analyses, including the chi-square test and the Mann–Whitney test [40], were performed using the Statistical Package for Social Sciences (SPSS, IBM; v25), with a cut-off level of statistical significance of *p* ≤ 0.05 [41]. The study was approved by the local institutional review board of the VUmc (2018.290). 

The time until the PSA progression was defined as the days between the surgery and the date of the BCR development. If no BCR was found, the maximum follow-up time was defined as the days between the surgery and the end-date of inclusion, 31 December 2018. Estimates of progression-free survival were derived using Kaplan–Meier estimates [42]. We evaluated whether statin users showed a different progression-free survival compared to non-statin users, and whether patterns of progression-free survival by statin use differed between the indicated risk groups. We defined statin use as any statin use (e.g., simvastatin, atorvastatin, rosuvastatin, pravastatin) within the inclusion period (2006–2018). 

This retrospective study was approved by the institutional review board of the Amsterdam University Medical Centers (VU Medical Center). The need for written informed consent was waived (review number 2018.290).

## 5. Conclusions

The use of statins did not seem to affect PCa development in a large cohort of patients with elevated levels of PSA. Statin use was not different between the different risk groups, indicating that statins do not affect PCa progression. Furthermore, the development of BCR after RP was not affected by the use of statins. This study had limited data on the length of statin exposure. Therefore, future prospective trials with controlled medications are essential in the interpretation of the effect of statin use on PCa. 

## Figures and Tables

**Table 1:** Characteristics and outcomes of each cohort.

Parameters	Prostate Cancer (n = 1122)	Control Group (n = 444)	*p*
Age, yrs; median (IQR)	66 (61–70)	64 (59–70)	0.24
Body Mass Index, kg/m^2^; mean (SD)	26.5 (3.6)	25.7 (4.3)	0.01
First PSA measurement; median (IQR)	9.9 (6.7–16.3)	6.4 (4.8–10.3)	0.93
Statin use, n (%)	252 (22.5)	83 (18.7)	0.10
Type of statins, n (%)			
Simvastatin	160 (63.5)	45 (54.2)	0.13
Atorvastatin	44 (17.5)	22 (26.5)	0.07
Rosuvastatin	22 (8.7)	7 (8.4)	0.93
Pravastatin	26 (10.3)	9 (10.8)	0.89
Gleason score, n (%)			
6	219 (19.5)		
7	661 (58.9)		
≥8	242 (21.6)		
Pathological T-stage, n (%)			
T2b or lower	104 (9.3)		
T2c or higher	533 (47.5)		
Missing	485 (43.2)		
ESMO-classification, n (%)			
Low Risk	103 (9.2)		
Intermediate Risk	588 (52.4)		
High Risk	431 (38.4)		
Surgery, n (%)			
Received prostate surgery	728 (64.9)		
No prostate surgery	394 (35.1)		
PSA-progression after surgery, n (%)			
No PSA-progression	365 (87.7)		
PSA-progression	51 (12.3)		
Follow-up in months; mean (SD)	50.0 (33.3)		

BMI: body mass index; PSA: prostate-specific antigen.

**Table 2:** Distribution of statin use and ESMO-classification per body mass index (BMI) group; Distribution of ESMO-classification among statin users.

**BMI**	**Statin User (n = 308)**	**Non-Statin User (n = 821)**	***p***
BMI < 25 kg/m^2^, n (%)	89 (28.9)	322 (39.2)	0.001
BMI < 30 kg/m^2^, n (%)	250 (81.1)	705 (85.9)	0.05
**ESMO-Classification, n (%)**	**BMI < 25 kg/m^2^, (n = 326)**	**BMI ≥ 25 kg/m^2^, (n = 618)**	***p***
Low Risk	18 (5.5)	33 (5.3)	0.91
Intermediate Risk	173 (53.1)	348 (56.3)	0.34
High Risk	135 (41.4)	237 (38.3)	0.36
**ESMO-Classification, n (%)**	**BMI < 30 kg/m^2^, (n = 796)**	**BMI ≥ 30 kg/m^2^, (n = 148)**	***p***
Low Risk	39 (4.9)	12 (8.1)	0.11
Intermediate Risk	447 (56.2)	74 (50.0)	0.17
High Risk	310 (38.9)	62 (41.9)	0.50
**ESMO-Classification, n (%)**	**Statin User (n = 252)**	**Non-Statin User (n = 870)**	***p***
Low Risk	22 (8.7)	81 (9.3)	0.78
Intermediate Risk	129 (51.2)	459 (52.8)	0.66
High Risk	101 (40.1)	330 (37.9)	0.54

BMI: body mass index.

**Table 3:** Overview of age, categorized per BMI and statin use.

Age, Yrs; Median (IQR)	Prostate Cancer	Control Group	*p*
BMI < 30 kg/m^2^	66 (61–70)	66 (60–72)	0.20
BMI ≥ 30 kg/m^2^	66 (61–70)	68 (63–70)	0.19
Statin use	68 (64–72)	68 (62–72)	0.37

BMI: body mass index.
